# A phase I study of nolatrexed dihydrochloride in children with advanced cancer. A United Kingdom Children's Cancer Study Group Investigation

**DOI:** 10.1054/bjoc.2000.1569

**Published:** 2001-01-01

**Authors:** E J Estlin, C R Pinkerton, I J Lewis, L Lashford, H McDowell, B Morland, J Kohler, D R Newell, A V Boddy, G A Taylor, L Price, S Ablett, R Hobson, M Pitsiladis, M Brampton, N Clendeninn, A Johnston, A D J Pearson

**Affiliations:** United Kingdom Children's Cancer Study Group, Department of Epidemiology and Public Health, UKCCSG Data Centre, University of Leicester, 22-28 Princess Road West, Leicester, LE1 6TP; Cancer Research Unit, Medical School, University of Newcastle, Newcastle upon Tyne, NE2 4HH; Cancer Research Campaign Phase I/II Clinical Trials Committee, Cancer Research Campaign, 10 Cambridge Terrace, Regent's Park, London, NW1 4JL; Agouron Pharmaceuticals Inc., 10350 North Torrey Pines Road, La Jolla, California, 92037-1020, USA

**Keywords:** acute lymphoblastic leukaemia, methotrexate resistance, pharmacokinetics, deoxyuridine

## Abstract

A phase I study of nolatrexed, administered as a continuous 5 day intravenous infusion every 28 days, has been undertaken for children with advanced malignancy. 16 patients were treated at 3 dose levels; 420, 640 and 768 mg/m^2^ 24 h^−1^. 8 patients were evaluable for toxicity. In the 6 patients treated at 768 mg/m^2^ 24 h^−1^, dose-limiting oral mucositis and myelosuppression were observed. Plasma nolatrexed concentrations and systemic exposure, measured in 14 patients, were dose related, with mean AUC values of 36 mg^−1^ ml^−1^ min^−1^, 50 mg ml^−1^ min^−1^ and 80 mg ml^−1^ min^−1^at the 3 dose levels studied. Whereas no toxicity was encountered if the nolatrexed AUC was <45 mg ml^−1^ min^−1^, Grade 3 or 4 toxicity was observed with AUC values of >60 mg ml^−1^ min^−1^. Elevated plasma deoxyuridine levels, measured as a surrogate marker of thymidylate synthase inhibition, were seen at all of the dose levels studied. One patient with a spinal primitive neuroectodermal tumour had stable disease for 11 cycles of therapy, and in two patients with acute lymphoblastic leukaemia a short-lived 50% reduction in peripheral lymphoblast counts was observed. Nolatrexed can be safely administered to children with cancer, and there is evidence of therapeutic activity as well as antiproliferative toxicity. Phase II studies of nolatrexed in children at the maximum tolerated dose of 640 mg/m^2^ 24 h^−1^are warranted. © 2001 Cancer Research Campaign http://www.bjcancer.com


British Journal of Cancer (2001) **84**(1), 11–18
© 2001 Cancer Research Campaign
doi: 10.1054/ bjoc.2000.1569, available online at http://www.idealibrary.com on IDEAL®

### A phase I study of nolatrexed dihydrochloride in children with advanced cancer. A United Kingdom Children's Cancer Study Group Investigation

**EJ Estlin[1], CR Pinkerton[1], IJ Lewis[1], L Lashford[1], H McDowell[1], B Morland[1], J Kohler, DR Newell[2], AV Boddy[2], GA Taylor[2], L Price[1], S Ablett[1], R Hobson[1], M Pitsiladis[3], M Brampton[3], N Clendeninn[4], A Johnston[4] and ADJ Pearson[1]**

[1]United Kingdom Children's Cancer Study Group, UKCCSG Data Centre, Department of Epidemiology and Public Health, University of Leicester, 22-28 Princess Road West, Leicester LE1 6TP; [2]Cancer Research Unit, Medical School, University of Newcastle, Newcastle upon Tyne, NE2 4HH; [3]Cancer Research Campaign Phase I/II Clinical Trials Committee, Cancer Research Campaign, 10, Cambridge Terrace, Regent's Park, London NW1 4JL. [4]Agouron Pharmaceuticals Inc., 10350 North Torrey Pines Road, La Jolla, California 92037-1020, USA

**Summary** A phase I study of nolatrexed, administered as a continuous 5 day intravenous infusion every 28 days, has been undertaken for children with advanced malignancy. 16 patients were treated at 3 dose levels; 420, 640 and 768 mg/m$^2$ 24 h$^{-1}$. 8 patients were evaluable for toxicity. In the 6 patients treated at 768 mg/m$^2$ 24 h$^{-1}$, dose-limiting oral mucositis and myelosuppression were observed. Plasma nolatrexed concentrations and systemic exposure, measured in 14 patients, were dose related, with mean AUC values of 36 mg$^{-1}$ ml$^{-1}$ min$^{-1}$, 50 mg ml$^{-1}$ min$^{-1}$ and 80 mg ml$^{-1}$ min$^{-1}$ at the 3 dose levels studied. Whereas no toxicity was encountered if the nolatrexed AUC was <45 mg ml$^{-1}$ min$^{-1}$, Grade 3 or 4 toxicity was observed with AUC values of >60 mg ml$^{-1}$ min$^{-1}$. Elevated plasma deoxyuridine levels, measured as a surrogate marker of thymidylate synthase inhibition, were seen at all of the dose levels studied. One patient with a spinal primitive neuroectodermal tumour had stable disease for 11 cycles of therapy, and in two patients with acute lymphoblastic leukaemia a short-lived 50% reduction in peripheral lymphoblast counts was observed. Nolatrexed can be safely administered to children with cancer, and there is evidence of therapeutic activity as well as antiproliferative toxicity. Phase II studies of nolatrexed in children at the maximum tolerated dose of 640 mg/m$^2$ 24 h$^{-1}$ are warranted. © 2001 Cancer Research Campaign http://www.bjcancer.com

**Keywords**: acute lymphoblastic leukaemia; methotrexate resistance; pharmacokinetics; deoxyuridine

Nolatrexed dihydrochloride (AG337, THYMITAQ™; Agouron Pharmaceuticals, Inc., San Diego, CA; Figure 1) is a novel folate-based inhibitor of thymidylate synthase (TS; EC 2.1.1.45). TS catalyses the terminal, rate limiting step of the de novo synthesis of thymidine nucleotides and thymidine nucleotides are used exclusively in the synthesis of DNA. TS is therefore an important target for antiproliferative cancer chemotherapy (Touroutoglou and Pazdur, 1996), and a number of classical antifolate TS inhibitors have been developed which have demonstrated activity in clinical trials in adults (Jackman and Calvert, 1995; Rustum et al, 1997). The α and γ glutamate carboxyl groups of classical antifolates, such as raltitrexed (an inhibitor of TS) and methotrexate (MTX), are negatively charged at physiological pH and thus require carrier mediated uptake for entry into cells (Goldman et al, 1968; Jansen et al, 1993). Once inside the cell, classical antifolates can undergo polyglutamation in a reaction catalysed by folylpolyglutamate synthetase (FPGS), which involves the addition of further glutamate residues at the γ-carboxyl position of the glutamate moiety (McGuire et al, 1980).

*Received 9 June 2000*
*Revised 22 September 2000*
*Accepted 11 October 2000*

*Correspondence to:* EJ Estlin

Polyglutamation enhances the intracellular retention and potency of classical antifolates against target enzymes such as TS (Allegra et al, 1985; Ward et al, 1992). However, resistance due to reduced cellular uptake or reduced formation of intracellular polyglutamates may limit the clinical activity of classical antifolate drugs. For example, the ability of lymphoblasts to metabolize MTX to long-chain polyglutamate forms may be an important determinant of the sensitivity childhood ALL to methotrexate-based therapy (Whitehead et al, 1992; Masson et al, 1996), and acquired resistance to MTX may be mediated by reduced transport and increased expression of dihydrofolate reductase (Gorlick et al, 1997). Nolatrexed, which as a nonclassical antifolate does not possess a terminal glutamate moiety, was rationally designed using X-ray crystallographic techniques (Webber et al, 1993) to overcome resistance to classical antifolates as a result of reduced uptake and polyglutamation.

Nolatrexed is a non-competitive TS inhibitor with an inhibition constant of 11 nM, with activity against rodent and human tumour cell lines in vitro, and against selected tumour models in vivo (Webber et al, 1996). Phase I studies in adults of nolatrexed administered either as a 5-day continuous infusion (Rafi et al, 1995), or orally every 6 hours for 5 days (Rafi et al, 1996), demonstrated dose-limiting myelosuppression with oral mucositis and a recommended Phase II dose of 800 mg/m$^2$ 24 h$^{-1}$. Evidence of clinical activity was observed when nolatrexed was administered as a 5 day intravenous infusion in Phase II studies in adults with

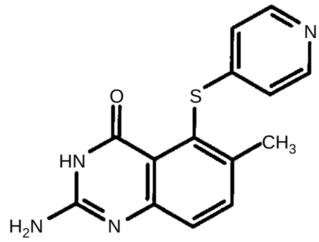

**Figure 1** Structure of nolatrexed hepatocellular carcinoma (Stuart et al, 1996), or squamous cell carcinoma of the head and neck (Belani et al, 1997).

To facilitate the rational introduction of nolatrexed into paediatric oncology practice, a Phase I study was performed. The aims of this Phase I study were to determine the maximally tolerated dose (MTD), dose-limiting toxicities and pharmacokinetics of nolatrexed, administered as a 5-day continuous intravenous infusion in children with advanced malignancy. In addition, evidence of TS inhibition was sought, by measurement of plasma UdR, and the plasma concentrations of nolatrexed associated with toxicity, efficacy and TS inhibition determined.

### METHODS

### Patient eligibility

The study was open from September 1995 until April 1998. Patients aged less than 18 years with histologically proven malignancy, including acute leukaemia and solid tumours involving the bone marrow, and for whom no conventional therapies were appropriate, were eligible. Patients were required to have a Lansky Performance Scale score of ≥30 (Lansky et al, 1987), but children were ineligible if they were a poor medical risk because of non-malignant systemic disease or uncontrolled infection, or had received chemotherapy or biologic therapy within 4 weeks of receiving nolatrexed. In cases of acute leukaemia which were refractory to chemotherapy, nolatrexed could be administered within 4 weeks of prior therapy, provided any toxicity associated with the previous treatment had resolved. Patients who had received radiation therapy within 6 weeks of receiving nolatrexed, or had concurrent malignancies at other sites were ineligible.

Laboratory investigations were performed within the 14 day period prior to the first dose of nolatrexed in order to ensure adequate haematological (Hb >9 g $dl^{-1}$, neutrophil count $>1 \times 10^9\ l^{-1}$, platelet count $>100 \times 10^9\ l^{-1}$), renal (serum creatinine ≤1.5 × the upper limit of laboratory normal for age) and hepatic (serum total bilirubin within the upper limit of laboratory normal, aspartate transaminase (AST) or alanine transaminase (ALT) ≤2 times the upper limit of laboratory normal) function. Patients with leukaemia or solid tumours with >25% bone marrow involvement were not required to fulfil the above haematological criteria. As well as baseline haematological and biochemical investigations, a full physical examination, electrocardiogram, urinalysis and radiological imaging of any tumour sites were performed within 14 days before the first dose of nolatrexed. In addition, for children with acute leukaemia or solid tumour with likely bone marrow involvement, a bone marrow aspirate and trephine were required. Patients were expected to have a life expectancy of at least 9 weeks, and written, informed and witnessed consent was obtained from all patients or their parents. The study was approved by the relevant local Ethics Committee.

### Study administration and drug supply

Patients were entered into the study from 7 participating United Kingdom Children's Cancer Study Group (UKCCSG) centres. Pre-registration (to determine availability of places), registration, requests for nolatrexed, adverse event reporting and notification of withdrawal from the study was co-ordinated by the UKCCSG Data Centre. The study was performed to standards equivalent to Good Clinical Practice guidelines (CPMP Working Party, 1990), and data were monitored and managed by the Cancer Research Campaign Phase I/II Clinical Trials Committee.

Nolatrexed dihydrochloride was manufactured by Agouron Pharmaceuticals (La Jolla, California, USA) and was supplied in 5 ml ampoules containing 450 mg of nolatrexed lyophilized powder for injection, together with 563.5 mg of mannitol which was used as an inactive caking agent, by the CRC Formulation Unit, Department of Pharmaceutical Sciences, University of Strathclyde, Glasgow, UK.

### Patient treatment and evaluation

Nolatrexed was administered by continuous intravenous infusion via a central line for 5 consecutive days. Anti-emetics were administered following the first episode of emesis, and were then allowed on a prophylactic basis. Treatment cycles were repeated every 28 days, provided there was recovery from toxicity. All responding patients, or those with stable disease, were allowed to continue on therapy with nolatrexed for up to one year at the discretion of their physician, unless disease progression was observed.

Patient review, physical examination, and measurement of the full blood count and serum concentrations of urea, creatinine, electrolytes, calcium, phosphate, magnesium, glucose, total bilirubin, total protein, albumin, alanine aminotransferase, alkaline phosphatase, were carried out weekly. Patients were evaluable for response if they received 5 days of nolatrexed, and were evaluable for toxicity if they survived for at least a further 23 days. Radiological investigations such as CT, MRI and ultrasonography were used to evaluate malignant lesions provided that at least one diameter was equal to or greater than 3 cm. Disease was measured in accordance with WHO criteria (WHO, 1979).

### Dose escalation

Nolatrexed doses were based on body surface area, which was calculated according to the formula recommended by Mosteller (1987)

$$\sqrt{\frac{\text{Height (cm)} \times \text{Weight (kg)}}{3600}}$$

Patients were recruited in cohorts of 3 per dose level, and dose-limiting toxicity was defined on the basis of toxicities observed on cycle 1 for each patient. No within-patient dose escalations were allowed. The initial dose level of 480 mg/$m^2$ 24 $h^{-1}$, which represented 60% of the adult MTD (Rafi et al, 1998), was chosen as prolonged dose-limiting myelosuppression and oral mucositis had been observed in a hevily pre-treated 18-year-old female with metastatic rhabdomyosarcoma, who had received nolatrexed on a compassionate use basis at the adult MTD. In addition, plasma

 © 2001 Cancer Research Campaign deoxyuridine levels were consistently elevated in adult patients receiving nolatrexed at 480 mg/m$^2$ 24 h$^{-1}$, indicating that systemic inhibition of the target enzyme was being achieved (Rafi et al, 1995).

### Definition of DLT and MTD

Dose-limiting toxicity was defined as a Serious Adverse Event that occurred during the first 28 days after the start of the nolatrexed infusion. The severity of toxicities were graded according to the Common Toxicity Criteria (CTC), and toxicity was considered to be dose limiting as follows:

#### *Non-haematological DLT*

Any Grade 3 or 4 toxicity, except for Grade 3 nausea and vomiting, Grade 3 hepatic toxicity which returned to a minimum of Grade 1 within to 28 days of nolatrexed administration, or Grade 3 fever. For mucositis to be dose limiting, Grade 4 toxicity was required to persist for at least 7 days.

#### *Haematological DLT (solid tumours with no or <25% bone marrow involvement)*

DLT was defined by CTC Grade 3 or 4 neutropenia, or Grade 3 thrombocytopenia lasting at least 7 days, or by Grade 4 thrombocytopenia of any duration.

#### *Haematological DLT (leukaemia and solid tumours with >25% bone marrow infiltration)*

DLT was defined by CTC Grade 4 neutropenia or thrombocytopenia with confirmatory bone marrow appearances of tri-lineage hypoplasia 5 weeks after receiving nolatrexed. DLT was defined by bone marrow aplasia which persisted for >5 weeks following the commencement of the nolatrexed cycle, as nadir neutrophil/platelet counts would not be relevant due to the underlying haemopoietic disorder (Ettinger et al, 1993).

When DLT was encountered in one patient of a cohort of 3, a maximum of 3 additional patients were treated at that level. If DLT was not observed with the additional patients, patients were entered at the next dose level. The MTD was defined as that dose level immediately below that at which a minimum of 2 patients in a cohort of 3 to 6 patients experienced DLT. A maximum of 1 out of 6 patients could experience DLT at the MTD dose level. A total of 6 patients were treated at the MTD dose level to fully assess toxicity, and this strategy was designed to allow the MTD to be estimated using a minimum number of patients (Korn et al, 1994).

### Pharmacokinetic and pharmacodynamic analyses

For the measurement of plasma nolatrexed, 3–5 ml blood samples were taken at 0, 1, 2, 3, 4, 6, 8 and 10 hours following the start of the 5 day infusion, daily (twice daily if resident in hospital) during the remainder of the infusion and at 0, 0.5, 1, 1.5, 2, 4, 6, 8, 10 and 24 hours after the end of the infusion. Daily 5 ml blood samples were also taken for plasma deoxyuridine analyses. All blood samples were taken into 10 ml lithium-heparin tubes to prevent coagulation, and the plasma fraction separated within 30 minutes of collection by centrifugation at 500 ***g***. Plasma samples were then stored at -20˚C until analysis. Urine samples were also collected, depending on the age of the child, for the measurement of the urinary excretion of nolatrexed. A baseline urine sample was collected, followed by 24 hour collections of urine during each day of the infusion and for 24 h after completion of the 5 day infusion. The volume of each 24 hour urine collection was recorded, and a 10 ml aliquot of urine taken and frozen at -20˚C until analysis.

Plasma and urinary nolatrexed concentrations, and plasma deoxyuridine concentrations were measured by the high performance liquid chromatography techniques as described previously (Rafi et al, 1995). Plasma pharmacokinetics were analysed by non-model dependent methods, again as previously described.

### RESULTS

### Patient characteristics

16 patients (7 male and 9 female) with a mean age of 8.5 years (range 1.5–17) were entered onto the study, and their details are listed in Table 1. The majority of children suffered from either

**Table 1** Patient characteristics II

| Unique Patient Number | Dose level (mg/m$^2$/24 hours) | Sex | Age (years) | Underlying disease | Evaluable for toxicity | Number of courses received |
|---|---|---|---|---|---|---|
| #01 | 480 | F | 4 | Non-Hodgkins lymphoma | No | 1 |
| #02 | 480 | F | 14 | ALL | No | 1 |
| #03 | 480 | M | 11 | ALL | No | 1 |
| #04 | 640 | M | 10 | AML | No | 1 |
| #05 | 640 | M | 8 | ALL | Yes | 1 |
| #06 | 640 | F | 1 | AML | No | 1 |
| #07 | 640 | M | 6 | AML | Yes | 1 |
| #08 | 640 | M | 4 | Neuroblastoma[b] | No | 1 |
| #09 | 640 | F | 17 | Osteogenic sarcoma | Yes | 2 |
| #10 | 640 | F | 3 | Peripheral Neuroectodermal tumour | Yes | 11 |
| #11 | 768 | F | 6 | Rhabdomyosarcoma[b] | Yes | 2 |
| #12 | 768 | M | 14 | ALL | No | 1[a] |
| #13 | 768 | F | 4 | Wilms' tumour | Yes | 1 |
| #14 | 768 | M | 11 | ALL | Yes | 1 |
| #15 | 768 | F | 7 | ALL | No | 1[a] |
| #16 | 768 | F | 16 | ALL | Yes | 1 |

[a] Indicates that the patient did not complete the 5 day intravenous infusion of nolatrexed. [b] Indicates disease involving the bone marrow.

*© 2001 Cancer Research Campaign*

**Table 2** Significant antiproliferative toxicities encountered following treatment with nolatrexed

| Dose level (mg/m² 24 h⁻¹) | Number entered | Evaluable for toxicity (*n*) | Patient identification | Maximum CTC Grade: Haematological | Maximum CTC Grade: Oral mucositis |
|---|---|---|---|---|---|
| 480 | 3 | None | | None | None |
| 640 | 7 | 4 | #05 | | 2 |
| | | | #09 | 3 | 3 |
| | | | #10 | 3 | 3 |
| 768 | 6 | 4 | #13 | 4 | 2 |
| | | | #14 | 4 | |
| | | | #16 | | 4 |

multiply relapsed acute lymphoblastic leukaemia ($n$ = 7) or acute myeloid leukaemia ($n$ = 3). All patients entering the study had received prior chemotherapy both at initial presentation and for at least their first relapse; 8 patients had received prior radiotherapy, 7 patients had received prior surgery and 2 patients had received a prior allogenic bone marrow transplantation.

### Dose escalation and toxicities

Of the 16 patients who entered the study, 8 completed the first 28 days required for toxicity evaluation. 4 patients (1 with non-Hodgkins lymphoma, 1 with ALL and 2 with AML) died of disease progression within the first cycle of therapy. Two patients with ALL were withdrawn from the study in order to receive alternative therapy, 1 patient experienced a Grade 4 anaphylactic reaction to the nolatrexed infusion and 1 patient was withdrawn from study during the toxicity evaluation period at parental request. Of the 3 patients who entered at the 480 mg/m$^2$ 24 h$^{-1}$ dose level, 2 patients with ALL were withdrawn on days 12 and 14, respectively, in order to receive alternative therapy. The third patient at the first dose level died of overwhelming progression of a Ki-1 positive NHL on day 16. However, no acute toxicities had been encountered in this patient cohort, and the plasma pharmacokinetic findings were in keeping with those in the adult Phase I study at a comparable dose level (see below). Therefore, since all acute antiproliferative toxicities were experienced within 14 days for adults receiving nolatrexed (Rafi et al, 1998), a decision to dose-escalate to the second dose level of 640 mg/m$^2$ 24 h$^{-1}$ was made.

Antiproliferative toxicities were encountered at the second and third dose levels (Table 2). At the 640 mg/m$^2$ 24 h$^{-1}$ dose level, patient #05 experienced Grade 2 oral mucositis from days 2 to 7. Patient #09 received 2 cycles of therapy with nolatrexed, and experienced Grade 3 oral mucositis from day 3 to 10 of each cycle, and Grade 3 thrombocytopenia and Grade 3 neutropenia during each cycle. At the 768 mg/m$^2$ 24 h$^{-1}$ dose level, toxicities became dose limiting. In patient #13, myelosuppression was dose limiting, with Grade 4 thrombocytopenia observed on day 7, which recovered to Grade 3 by day 9. This patient also experienced dose-limiting hepatic toxicity, with Grade 4 hyperbilirubinaemia observed on day 11. The bilirubin remained elevated throughout the duration of the first cycle. However, this patient was known to have metastatic disease in the area of the porta hepatis and had experienced previous liver dysfunction in response to therapy with actinomycin D. Two patients experienced dose-limiting oral mucositis. Patient #14 experienced Grade 4 oral mucositis, which began on day 2, for a duration of 7 days. Patient #16 experienced Grade 4 oral mucositis, which began on day 5, for a duration of 7 days. Patient #15 had an immediate Grade 4 anaphylactic reaction to the nolatrexed infusion and was withdrawn from study.

Patient #10 experienced Grade 3 oral mucositis from days 4 to 7, and Grade 3 neutropenia between day 7 and day 9 during the first cycle. This patient received 11 courses of therapy with nolatrexed, and experienced a changing pattern of antiproliferative toxicity with time. Whereas for the first 5 cycles of treatment Grade 2/3 mucositis and myelosuppression was encountered, these toxicites were less marked during latter cycles. Hyperbilirubinaemia, occurring with a median onset of 13 days (range 2–21 days) time was observed in 9 children and was dose limiting in one child. Elevations in hepatic transminases were reported in 9 patients (median onset of 6 days; range 1–21 days), but were not dose limiting.

A wide variety of other adverse events were reported, and these mainly reflected the poor medical condition of children with advanced malignancy. 6 children (38%) experienced ≤ Grade 2 nausea and/or vomiting during the period of the nolatrexed infusion, which was easily controlled with standard anti-emetic therapy. Other reported adverse events included electrolyte disturbances, constipation, pyrexia, fever and infection; however, these were felt to be unrelated to therapy with nolatrexed. The occurrence of a rash was reported in 36% of cases, varying from lymphomatous skin involvement and thrombocytopenic petechiae, to non-specific erythematous appearances. Renal impairment was reported in one patient (#12), a 14-year-old male with known tuberose sclerosis and multiply relapsed ALL. The patient developed convulsions, drowsiness, fever and plasma biochemical signs of tumour lysis with an elevated urate (× 2 the upper limit of normal), phosphate (Grade 2) and creatinine (Grade 2) on the second day of the nolatrexed infusion. The patient had a known past medical history of epilepsy, and the biochemical changes were not sufficient to warrant intervention with dialysis. The patient died on day 5, and a combination of leukaemia and infection was thought to be the reason for death.

### Pharmacokinetics

Plasma nolatrexed concentration-*versus*-time data were available for 14 patients. Steady-state plasma concentrations were generally achieved within 12 hours of the start of the infusion (Figure 2). Peak plasma levels ranged from 4 µg ml$^{-1}$ at 480 mg/m$^2$ 24 h$^{-1}$ to 20 µg ml$^{-1}$ at the 768 mg/m$^2$ 24 h$^{-1}$ dose level, and a 2–3 fold inter-individual variation in steady-state levels was observed at each dose level. For patients #9 and #13, there was a tendency for plasma levels to increase throughout the infusion period, suggesting saturation of nolatrexed clearance. A marked example of this apparent saturation was observed in patient #12, where an incomplete pharmacokinetic analysis was performed as the patient died on day 5 of the study. In this patient, the plasma nolatrexed concentrations achieved were the highest in this paediatric population; the patient having achieved an AUC of 88 mg ml$^{-1}$ min$^{-1}$ by 76 hours of an 83-hour infusion. Evidence of non-linearity with saturation of nolatrexed clearance with increasing dose level may be indicated by the relative increase in mean AUC. Whereas the 33% increase in nolatrexed dose between dose level one and dose level two was associated with a 38% increase in mean AUC, the

 © 2001 Cancer Research Campaign

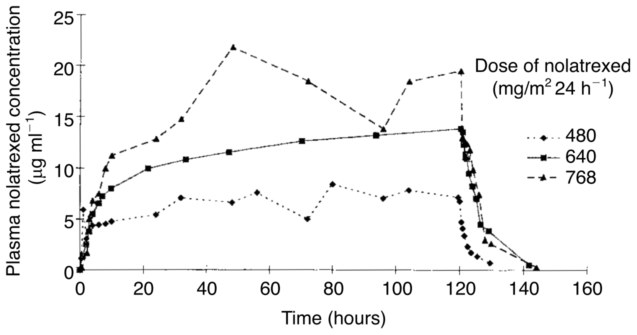

**Figure 2** Representative plasma nolatrexed concentration versus time data during and following a 5 day intravenous nolatrexed infusion at each dose level. Each data set represents an individual patient course

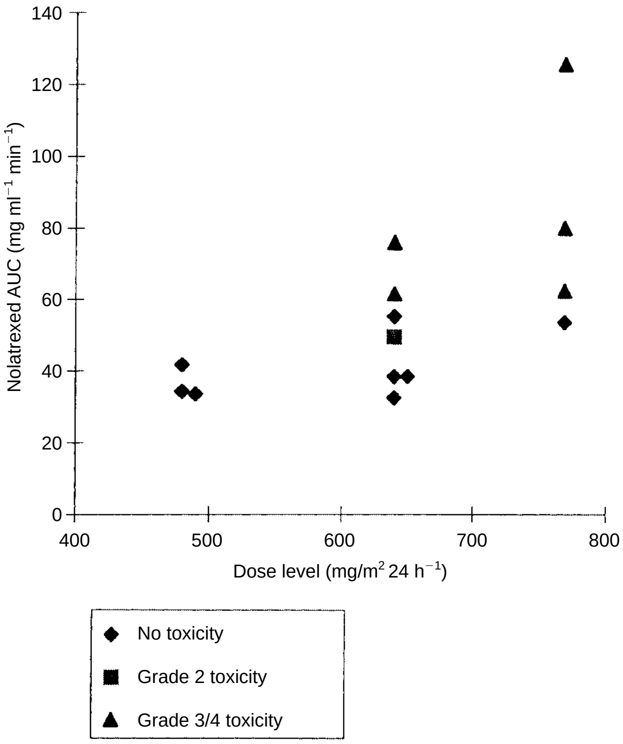

**Figure 3** Relationship between nolatrexed AUC and antiproliferative toxicity. Antiproliferative toxicities were those of oral mucositis and myelosuppression and related to the course of nolatrexed therapy on which pharmacokinetics were studied

20% increase in nolatrexed dose between dose level two and dose level three was associated with a 60% increase in mean AUC. Overall, across the three dose levels, a 60% increase in nolatrexed dose was associated with a 122% increase in mean AUC, and mean clearance values tended to decrease at the highest dose level.

The average systemic exposure to nolatrexed increased with increasing dose level ($r^2 = 0.4$, $P < 0.02$), although a 2–3 fold inter-individual variation in AUC was seen at the higher two dose levels (Figure 3). Nolatrexed was rapidly cleared from the plasma with a median elimination half-life of 1.9 hours (range 1.2 to 4.6 hours). Less than 30% of the nolatrexed dose was eliminated as unchanged drug in the urine (range 13–27%). Pharmacokinetic analyses are presented in Table 3.

### Responses

Short-lived oncolytic responses were observed in 2 patients with ALL. For patient #02, a reduction in the peripheral white cell count from $51 \times 10^9\ l^{-1}$ to $19 \times 10^9\ l^{-1}$ occurred by the end of the 5 day infusion. For patient #03, a reduction in the peripheral white cell count from $14 \times 10^9\ l^{-1}$ to $7 \times 10^9\ l^{-1}$ was observed. However, both patients were withdrawn from study by day 14 to receive alternative therapy with methylprednisolone. For a patient with a peripheral neuroectodermal tumour (patient #10), stable disease was observed for 11 months.

### Pharmacokinetic/pharmacodynamic relationships

The pharmacodynamic parameters studied following nolatrexed administration were antiproliferative toxicity (mucositis and myelosuppression) and nolatrexed-induced changes in plasma deoxyuridine concentrations. As shown in Figure 3, whereas no toxicity was encountered if the nolatrexed AUC was < 45 mg $ml^{-1}$ $min^{-1}$, Grade 3 or 4 toxicity was observed in all 5 patients who achieved an AUC of > 60 mg $ml^{-1}$ $min^{-1}$. There was no relationship between the occurrence of hyperbilirubinaemia and nolatrexed dose-level or AUC.

The effect of nolatrexed administration on plasma UdR concentrations was studied in 3 children at each dose level. Plasma UdR concentrations were elevated within 24 hours of the beginning of the nolatrexed infusion; however, a 6-fold variation in pretreatment plasma UdR levels was observed within this paediatric population. Similarly substantial inter-individual variation in the magnitude of the UdR elevation was found (Figure 4). As shown in Figure 4, there was no nolatrexed dose dependency in the elevation of plasma UdR levels, and no evidence of a relationship between UdR elevation and antiproliferative toxicity.

**Table 3** Pharmacokinetic parameters for nolatrexed administered by 5-day intravenous infusion

| Dose level (mg/m² 24 h⁻¹) | *n* | AUC (mg ml⁻¹ min⁻¹) | | Volume of distribution (l/m²) | | Clearance (l/m² h⁻¹) | | $t_{1/2}$ (hours) | | Urine excretion (%) | |
|---|---|---|---|---|---|---|---|---|---|---|---|
| | | Mean | Range | Mean | Range | Mean | Range | Mean | Range | Mean | Range |
| 480 | 3 | 36 | 33–42 | 9 | 7–10 | 4 | 3.4–4.3 | 1.6 | 1.2–1.9 | 15 | 14–18 |
| 640 | 7 | 50 | 32–76 | 14 | 7–22 | 4.1 | 2.3–5.9 | 2.6 | 1.4–4.6 | 15 | 13–16 |
| 768 | 4 | 80 | 53–125 | 9 | 7–12 | 3.2 | 1.8–4.3 | 2.2 | 1.23–2.8 | 19 | 12–27 |

Parameters were calculated using model-independent pharmacokinetic analyses as described in methods.

© 2001 Cancer Research Campaign

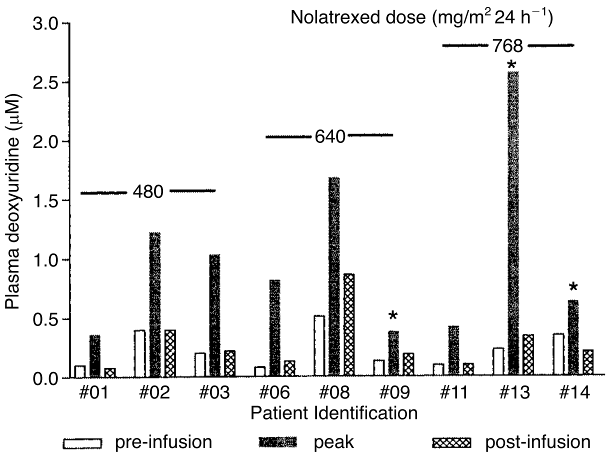

**Figure 4** Plasma deoxyuridine concentrations in children treated with nolatrexed. Each triplet of bars represents data from an individual patient course. Data marked with an asterisk (*) were those courses associated with a Grade 3/4 antiproliferative toxicity.

### DISCUSSION

This paper describes a Phase I study of nolatrexed, administered as a 5-day continuous intravenous infusion, in children with advanced cancer. The primary aims of the study were to determine the MTD, dose-limiting toxicities and pharmacokinetics of nolatrexed in children with advanced cancer, together with the characterization of pharmacokinetic–pharmacodynamic relationships. A secondary aim was to document antitumour efficacy.

The study was initially designed to be performed solely in children with acute leukaemia or solid tumours with >25% bone marrow involvement. However, this population of patients are both heavily pre-treated and have multiply drug-resistant end stage disease. In addition, we had elected to commence recruitment at 480 mg/m$^2$ 24 h$^{-1}$, and assess the tolerability of nolatrexed in children at this dose level, in view of the adult experience of toxicity in a heavily pretreated 18 year old patient receiving 640 mg/m$^2$ 24 h$^{-1}$ nolatrexed on compassionate grounds. Therefore, dose-escalation from the first dose level, which represented 60% of the adult MTD, was undertaken even though the first 3 patients recruited to the study did not strictly satisfy the criteria for toxicity evaluation. However, for all these patients, acute toxicities were not observed in the first 14 days of follow up, during which time acute toxicity would be observed in adults receiving nolatrexed (Rafi et al, 1998), and the plasma pharmacokinetic analyses were in keeping with the adult Phase I study of nolatrexed. However, of the first 8 patients who were entered into the study, only 2 satisfied the requirements for toxicity evaluation, i.e. to remain on study for 28 days without receiving therapy such as high-dose corticosteroids. Therefore, the eligibility criteria of the study were broadened to include all children with advanced cancer, which improved the rate of subsequent patient recruitment and successful toxicity evaluation.

The dose-limiting antiproliferative toxicities of oral mucositis and myelosuppression observed with nolatrexed in children are in keeping with the toxicity findings of the adult Phase I study of the drug when administered as a 5-day intravenous infusion (Rafi et al, 1998). However, the MTD defined by the current study, i.e. 640 mg/m$^2$ 24 h$^{-1}$, is 80% of the recommended adult Phase II dose determined by the adult Phase I study. This apparent discrepancy may reflect the more intensive prior therapy that this paediatric population had received. Indeed, although it has been recognized that a wide range in variation of the paediatric versus adult MTD exists for many antineoplastic agents (Marsoni et al, 1985) which can reflect differences in the intensity of prior chemotherapy, and/or physiological differences in renal function, hepatic metabolism and body composition (Balis et al, 1993), there has been a trend towards a lower paediatric:adult MTD ratio over the past decade, which illustrates the importance of prior therapy in the relative tolerance of new anticancer agents (Carlson et al, 1996). Furthermore, more recent Phase I studies have accrued a second stratum of less heavily pre-treated children (Blaney et al, 1997).

As in the adult Phase I study, the onset of oral mucositis occurred early in the course of therapy with nolatrexed, and was usually established before the end of the 5-day infusion. Other toxicities encountered in this paediatric Phase I study, such as nausea and vomiting, diarrhoea, rash and hepatotoxicity were similar to those reported in the adult 5-day intravenous Phase I study (Rafi et al, 1998). However, the focal desquamation of the hands, feet and scrotal areas observed in the adult study were not encountered in the present study. For the adult Phase I studies of nolatrexed administered as 5-day and 10-day intravenous infusions, thrombotic episodes in the vein receiving the nolatrexed infusion were reported (Creaven et al, 1998; Rafi et al, 1998). This complication was not recognized in the present paediatric study, although as in the adult 5- and 10-day studies, nolatrexed was administered via a central venous catheter. The major toxicities of oral mucositis and myelosuppression encountered in the present study are similar to those described in children for other antifolates such as MTX (Wolfram et al, 1993), trimetrexate (Balis et al, 1987) and piritrexim (Adamson et al, 1990).

In the adult 5-day intravenous study, a dose-dependent decrease in nolatrexed clearance was observed, in keeping with saturable elimination (Rafi et al, 1998). Moreover, at doses ≥768 mg/m$^2$ 24 h$^{-1}$, there was a tendency for plasma nolatrexed levels to increase throughout the infusion period. A similar finding was demonstrated in 3 children in the present study. Indeed, marked nonlinearity was observed in a patient with tuberose sclerosis and relapsed ALL, who was treated at the 768 mg/m$^2$ 24 h$^{-1}$ dose level in the present study. The reasons for this observation are not certain; there was no obvious mechanism for a drug interaction, and the finding may represent abnormally low hepatic metabolism, or reduced renal elimination in the face of impaired renal function secondary to tumour lysis. Although the mean nolatrexed AUC increased with successive dose levels in the present study, a 2-to 3-fold inter-individual variation was observed at each dose level. A similar inter-individual variation in systemic exposure was found for adults receiving nolatrexed (Rafi et al, 1998), and pharmacokinetic variability has been recognized for a variety of anticancer agents in children (Rodman et al, 1993). As with the adult 5-day intravenous study, increases in nolatrexed AUC were related to antiproliferative toxicity. Similarly, <30% of nolatrexed was excreted in the urine.

Measurement of plasma deoxyuridine concentrations indicated that systemic inhibition of TS occurred at each dose level in the present study. This is in keeping with the findings of the study of nolatrexed administered as a 24-hour infusion in adults (Rafi et al, 1995), where doses of nolatrexed at 480 mg/m$^2$ and above were associated with elevations of plasma deoxyuridine concentrations. Moreover, as for the Phase I study of nolatrexed administered as a 5-day continuous infusion in adults (Rafi et al, 1998), there was no

*© 2001 Cancer Research Campaign*

clear relationship between the increase in UdR concentrations and nolatrexed AUC or antiproliferative toxicity.

In conclusion, nolatrexed given as a 5-day continuous infusion in children produces predictable and reversible antiproliferative toxicities, and plasma biochemical evidence of systemic TS inhibition. The low rate of completion of toxicity evaluation for children with acute leukaemia highlights the difficulty of performing single agent Phase I studies in this patient population. Although antitumour activity with nolatrexed has been demonstrated in Phase II studies in adults, no objective responses were observed in the present study. This may not be surprising given the intensity of previous therapy experienced by this paediatric population. The further evaluation of this class of anticancer agents in children is warranted.

### ACKNOWLEDGEMENTS

The authors gratefully acknowledge the participation of the following UKCCSG centres during this study: The Royal Victoria Infirmary, Newcastle upon Tyne, NE1 4LP, UK; Royal Marsden Hospital, Sutton, Surrey, SM2 5PT, UK; St James University Hospital, Leeds, LS9 7TF, UK; Birmingham Children's Hospital, Birmingham, B4 6NH, UK; Christie Hospital NHS Trust, Manchester, M20 4BX, UK; Alder Hey Children's Hospital, Liverpool, L12 2AP, UK; and Southampton General Hospital, Southampton SO9 4XY, UK. The authors also gratefully acknowledge the Cancer Research Campaign Phase I/II Clinical Trials Committee, 10, Cambridge Terrace, London, NW1 4JL, UK; Agouron Pharmaceuticals Inc., La Jolla, California, USA, and Dr Gavin Halbert, Cancer Research Campaign Formulation Unit, University of Strathclyde, Glasgow, UK.

### REFERENCES

Adamson PC, Balis FM, Miser J, Wells RJ, Bleyer WA, Williams TE, Gillespie A, Penta JS, Clendeninn NJ and Poplack DG (1990) Pediatric Phase I trial and pharmacokinetic study of piritrexim administered orally on a 5-day schedule. *Cancer Res* **50**: 4464–4467

Allegra CJ, Drake JC, Jolivet J and Chabner BA (1985a) Inhibition of phosphoribosylaminoimadazolecarboxamide transformylase by methotrexate and dihydrofolic acid polyglutamates. *Proc Natl Acad Sci USA* **82**: 4881–4885

Allegra CJ, Chabner BA, Drake JC, Lutz R, Rodbard D and Jolivet J (1985b) Enhanced inhibition of thymidylate synthase by methotrexate polyglutamates. *J Biol Chem* **260**: 9720–9726

Balis FM, Patel R, Luks E, Doherty KM, Holcenberg JS, Tan C, Reaman GH, Belasco J, Ettinger LJ, Zimm S and Poplack DG (1987) Pediatric Phase I trial and pharmacokinetic study of trimetrexate. *Cancer Res* **47**: 4973–4976

Balis F, Holcenberg J and Poplack D (1993) General Principles of Chemotherapy. In: Pizzo P, Poplack D (ed) *Principles and Practice of Pediatric Oncology*, 2nd edition. pp 197–245. Philadelphia: JB Lippincott Company

Belani CP, Aqarwala S, Johnson T, Cohn A, Bernstein J, Langer C, Jones V, White C, Lob D, White D, Chew T, Johnston A, and Clendeninn N (1997) A phase II trial of Thymitaq™ in patients with squamous cell carcinoma of the head and neck. *Proc Am Assoc Cancer Res* **16**: 387a

Blaney SM, Seibel NL, O'Brien M, Reaman GH, Berg S, Adamson PC, Poplack DG, Krailo MD, Mosher R and Balis FM (1997) Phase I trial of docetaxel administered as a 1-hour infusion in children with refractory solid tumours: a Collaborative Pediatric Branch, National Cancer Institute and Children's Cancer Group Trial. *J Clin Oncol* **15**: 1538–1543

Carlson L, Ho P, Smith M, Reisch J and Weitman S (1996) Pediatric Phase I drug tolerance: a review and comparison of recent adult and pediatric Phase I trials. *J Ped Hematol Oncol* **18**: 250–256

CPMP Working Party on the efficacy of Medicinal Products (1990) European Community Commission notes for guidance: Good Clinical Practice for trials of Medicinal Products in the European Community. *J Pharmacol Toxicol* **67**: 361–372

Creaven PJ, Pendyala L, Meropol NJ, Clendeninn NJ, Wu EY, Loewen GM, Proefrock A, Johnston A and Dixon M (1998) Initial clinical trial and pharmacokinetics of Thymitaq™ (AG337) by 10-day continuous infusion in patients with advanced solid tumours. *Cancer Chemother Pharmacol* **41**: 167–170

Ettinger LJ, Krailo MD, Gaynon PS and Hammond GD (1993) A Phase I study of carboplatin in children with acute leukaemia in bone marrow relapse. A report from the Children's Cancer Group. *Cancer* **72**: 917–22

Goldman ID, Lichtenstein NS and Oliverio VT (1968) Carrier-mediated transport of the folic acid analogue, methotrexate, in the L1210 leukemia cell. *J Biol Chem* **243**: 5007–5017

Gorlick R, Goker E, Trippett T, Steinherz P, Elisseyeff Y, Mazumdar M, Flintoff WF and Bertino JR (1997) Defective transport is a common mechanism of acquired methotrexate resistance in acute lymphoblastic leukaemia and is associated with decreased reduced folate carrier expression. *Blood* **89**: 1013–1018

Jackman AL and Calvert AH (1995) Folate-based thymidylate synthase inhibitors as anticancer drugs. *Ann Oncol* **6**: 871–881

Jansen G, Westerhof GR, Schornagel JH, Jackman AL and Boyle FT (1993) The reduced folate methotrexate carrier and a membrane-associated folate binding protein as transport routes for novel antifolates: structure-activity relationships. In: Ayling JE (ed) *Chemistry and Biology of Pteridines and Folates*, pp 767–770. Plenum Press: New York

Korn EL, Midthune D, Chen TT, Rubinstein LV, Christian MC and Simon RM (1994) A comparison of two Phase I trial designs. *Statistics in Medicine* **13**: 1799–1806

Lansky SB, List MA, Lansky LL, Ritter-Sterr C and Miller DR (1987) The measurement of performance in childhood cancer. *Cancer* **60**: 1651–56

McGuire JJ, Hsiech P, Coward JK and Bertino JR (1980) Enzymatic synthesis of the folylpolyglutamates. *J Biol Chem* **255**: 5776–5788

Marsoni S, Ungerleider R, Hurson S and Hammershaimb L (1985) Tolerance to antineoplastic agents in children and adults. *Cancer Treat Rep* **69**: 1263–1269

Masson E, Relling MV, Synold TW, Liu Q, Scheutz JD, Sandlund JT, Pui C-H and Evans WE (1996) Accumulation of methotrexate polyglutamates in lymphoblasts is a determinant of antileukaemic effects in vivo. *J Clin Invest* **97**: 73–80

Mosteller RD (1987) Simplified calculation of body-surface area. *New Eng J Med* **317**: 1098

Rafi I, Taylor GA, Balmanno K, Calvete JA, Boddy AV, Bailey NB, Lind MJ, Calvert AH, Webber S, Jackson RC, Johnston A, Clendeninn N and Newell DR (1995) Clinical Pharmacokinetic and pharmacodynamic studies with the nonclassical antifolate thymidylate synthase inhibitor 3,4-dihydro-2-amino-6-methyl-4-oxo-5-(4-pyridylthio)-quinazolone dihydrochloride (AG337) given by 24-hour continuous intravenous infusion. *Clin Cancer Res* **1**: 1275–1284

Rafi I, Boddy AV, Taylor GA, Calvete JA, Bailey NB, Lind MJ, Newell DR and Calvert AH (1996) A Phase I clinical study of the novel antifolate AG337 given by 5 day oral administration. *Br J Cancer* **73** (suppl 26): 15

Rafi I, Boddy AV, Calvete JA, Taylor GA, Newell DR, Bailey NP, Lind MJ, Green M, Hines J, Johnston A, Clendeninn N and Calvert AH (1998) Preclinical and Phase I clinical studies with the nonclassical antifolate thymidylate synthase inhibitor nolatrexed dihydrochloride given by prolonged administration in patients with solid tumours. *J Clin Oncol* **16**: 1131–1141

Rodman JH, Relling MV, Stewart CF, Synold TW, McLeod H, Kearns C, Stute N, Crom WR and Evans WE (1993) Clinical pharmacokinetics and pharmacodynamics of anticancer drugs in children. *Semin Oncol* **20**: 18–29

Rustum YM, Harstick A, Cao S, Vanhoefer U, Yin M-B, Wilke H and Seeber S (1997) Thymidylate synthase inhibitors in cancer therapy: direct and indirect inhibitors. *J Clin Oncol* **15**: 389–400

Stuart KE, Hajdenberg J, Cohn A, Loh KK, Miller W, White C and Clendeninn NJ (1996) A Phase II trial of Thymitaq™ in patients with hepatocellular carcinoma. *Proc Am Assoc Clin Oncol* **15**: 202

Touroutoglou N and Pazdur R (1996) Thymidylate synthase inhibitors. *Clin Cancer Res* **2**: 227–243

Ward WHJ, Kimbell R and Jackman AL (1992) Kinetic characteristics of ICI D1694: a quinazoline antifolate which inhibits thymidylate synthase. *Biochem Pharmacol* **43**: 2029–2031

Webber SE, Bleckman TH, Attard J, Deal JG, Kathardekar V, Welsh KM, Webber S, Janson CA, Matthews DA and Smith WW (1993) Design of thymidylate synthase inhibitors using protein crystal structures: The synthesis and biological evaluation of a novel class of 5-substituted quinazolinones. *J Med Chem* **36**: 733–46

Webber S, Bartlett CA, Boritzki TJ, Hilliard JA, Howland EF, Johnston, AL, Kosa M, Margosiak SA, Morse CA and Shetty BV (1996) AG337, a novel lipophilic thymidylate synthase inhibitor: *in vitro* and *in vivo* preclinical studies. *Cancer Chemother Pharmacol* **37**: 509–517

© 2001 Cancer Research Campaign 

Whitehead VM, Vuchich MJ, Lauer SJ, Mahoney D, Carroll AJ, Schuster JJ, Esseltine DW, Payment C, Look AT, Akabutu J, Taylor LD, Camitta B and Pullen DJ (1992) Accumulation of high levels of methotrexate polyglutamates in lymphoblasts from children with hyperdiploid (> 50 chromosomes) B-lineage acute lymphoblastic leukaemia: a Pediatric Oncology Group Study. *Blood* **80**: 1316–1323

WHO handbook for reporting results of cancer treatment (1979) WHO offset publication. 48-World Health Organisation, Geneva.

Wolfram C, Hartmann R, Fengler R, Bruhmuller S, Ingwersen A and Henze G (1993) Randomised comparison of 36-hour intermediate-dose versus 4-hour high-dose methotrexate infusions for remission induction in relapsed childhood acute lymphoblastic leukaemia. *J Clin Oncol* **11**: 827–833

*© 2001 Cancer Research Campaign*
